# What Do We Have to Know about PD-L1 Expression in Prostate Cancer? A Systematic Literature Review. Part 2: Clinic–Pathologic Correlations

**DOI:** 10.3390/cells10113165

**Published:** 2021-11-14

**Authors:** Andrea Palicelli, Martina Bonacini, Stefania Croci, Cristina Magi-Galluzzi, Sofia Cañete-Portillo, Alcides Chaux, Alessandra Bisagni, Eleonora Zanetti, Dario De Biase, Beatrice Melli, Francesca Sanguedolce, Magda Zanelli, Maria Paola Bonasoni, Loredana De Marco, Alessandra Soriano, Stefano Ascani, Maurizio Zizzo, Carolina Castro Ruiz, Antonio De Leo, Guido Giordano, Matteo Landriscina, Giuseppe Carrieri, Luigi Cormio, Daniel M. Berney, Jatin Gandhi, Giacomo Santandrea, Maria Carolina Gelli, Alessandro Tafuni, Moira Ragazzi

**Affiliations:** 1 Pathology Unit, Azienda USL-IRCCS di Reggio Emilia, 42123 Reggio Emilia, Italy; Alessandra.Bisagni@ausl.re.it (A.B.); Eleonora.Zanetti@ausl.re.it (E.Z.); magda.zanelli@ausl.re.it (M.Z.); mariapaola.bonasoni@ausl.re.it (M.P.B.); loredana.demarco@ausl.re.it (L.D.M.); giacomo.santandrea@ausl.re.it (G.S.); MariaCarolina.Gelli@ausl.re.it (M.C.G.); alessandro.tafuni@unipr.it (A.T.); Moira.Ragazzi@ausl.re.it (M.R.); 2 Clinical Immunology, Allergy and Advanced Biotechnologies Unit, Azienda USL-IRCCS di Reggio Emilia, 42123 Reggio Emilia, Italy; Martina.Bonacini@ausl.re.it (M.B.); Stefania.Croci@ausl.re.it (S.C.); 3 Department of Pathology, University of Alabama at Birmingham, Birmingham, AL 35294, USA; cmagigalluzzi@uabmc.edu (C.M.-G.); scaneteportillo@uabmc.edu (S.C.-P.); 4 Department of Scientific Research, School of Postgraduate Studies, Norte University, Asunción 1614, Paraguay; alcideschaux@uninorte.edu.py; 5 Department of Pharmacy and Biotechnology (FABIT), University of Bologna, 40126 Bologna, Italy; dario.debiase@unibo.it; 6 Fertility Center, Department of Obstetrics and Gynecology, Azienda USL-IRCCS di Reggio Emilia, 42123 Reggio Emilia, Italy; Beatrice.Melli@ausl.re.it; 7 Clinical and Experimental Medicine PhD Program, University of Modena and Reggio Emilia, 41121 Modena, Italy; Carolina.CastroRuiz@ausl.re.it; 8 Pathology Unit, Policlinico Riuniti, University of Foggia, 71122 Foggia, Italy; francesca.sanguedolce@unifg.it; 9 Department of Pathology, Case Western Reserve University, Cleveland, OH 44106, USA; alessandra.soriano@ausl.re.it; 10 Gastroenterology Division, Azienda USL-IRCCS di Reggio Emilia, 42123 Reggio Emilia, Italy; 11 Pathology Unit, Azienda Ospedaliera Santa Maria di Terni, University of Perugia, 05100 Terni, Italy; s.ascani@aospterni.it; 12 Haematopathology Unit, CREO, Azienda Ospedaliera di Perugia, University of Perugia, 06129 Perugia, Italy; 13 Surgical Oncology Unit, Azienda USL-IRCCS di Reggio Emilia, 42123 Reggio Emilia, Italy; maurizio.zizzo@ausl.re.it; 14 Molecular Diagnostic Unit, Azienda USL Bologna, Department of Experimental, Diagnostic and Specialty Medicine, University of Bologna, 40138 Bologna, Italy; antonio.deleo@unibo.it; 15 Medical Oncology Unit, Department of Medical and Surgical Sciences, University of Foggia, 71122 Foggia, Italy; guido.giordano@unifg.it (G.G.); matteo.landriscina@unifg.it (M.L.); 16 Department of Urology and Renal Transplantation, University of Foggia, 71122 Foggia, Italy; giuseppe.carrieri@unifg.it (G.C.); luigi.cormio@unifg.it (L.C.); 17 Barts Cancer Institute, Queen Mary University of London, London EC1M 5PZ, UK; daniel.berney@nhs.net; 18 Department of Pathology and Laboratory Medicine, University of Washington, Seattle, WA 98195, USA; jgandhi@uw.edu; 19 Pathology Unit, Department of Medicine and Surgery, University of Parma, 43121 Parma, Italy

**Keywords:** PD-L1, prostate, cancer, adenocarcinoma, immunohistochemistry, target-therapy, immunotherapy, checkpoint inhibitors

## Abstract

Many studies have investigated the potential prognostic and predictive role of PD-L1 in prostatic carcinoma (PC). We performed a systematic literature review (PRISMA guidelines) to critically evaluate human tissue-based studies (immunohistochemistry, molecular analysis, etc.), experimental research (cell lines, mouse models), and clinical trials. Despite some controversial results and study limitations, PD-L1 expression by tumor cells may be related to clinic–pathologic features of adverse outcome, including advanced tumor stage (high pT, presence of lymph node, and distant metastases), positivity of surgical margins, high Grade Group, and castration resistance. Different PD-L1 positivity rates may be observed in matched primary PCs and various metastatic sites of the same patients. Over-fixation, type/duration of decalcification, and PD-L1 antibody clone may influence the immunohistochemical analysis of PD-L1 on bone metastases. PD-L1 seemed expressed more frequently by castration-resistant PCs (49%) as compared to hormone-sensitive PCs (17%). Some series found that PD-L1 positivity was associated with decreased time to castration resistance. Treatment with ipilimumab, cyclophosphamide/GVAX/degarelix, or degarelix alone may increase PD-L1 expression. Correlation of PD-L1 positivity with overall survival and outcomes related to tumor recurrence were rarely investigated; the few analyzed series produced conflicting results and sometimes showed limitations. Further studies are required. The testing and scoring of PD-L1 should be standardized.

## 1. Introduction

According to the “Surveillance, Epidemiology, and End Results Program” (SEER) database of the US National Cancer Institute, prostatic carcinoma (PC) accounted for 10.6% of all new cancer cases and 5.5% of all deaths for cancer worldwide from 2016 to 2018. A total of 191,930 new diagnoses were estimated in 2020, with 33,330 men dying from PC [1,2,3,4]. In higher-income countries, the estimated five-year survival rate is 97.8%, decreasing to 30% in the case of advanced disease [1]. Especially for this latter subgroup of patients, the progress of new strategies for treatment is an urgent need. As for other tumors, new cost-effective diagnostic, prognostic, and treatment-predictive biomarkers have been increasingly tested in PC [2,3,4,5,6,7].

The potential role of immune-checkpoint therapy targeting the PD-1/PD-L1 axis has been investigated in different tumors, sometimes obtaining promising results [8]. As regards PC patients, an anti-PD-1 antibody (pembrolizumab) recently revealed promising therapeutic activity in a phase II trial, and the 2021 United States National Comprehensive Cancer Network (NCCN) guidelines have allowed its administration in selected cases [4,9].

Some studies have investigated the prognostic significance of PD-L1 expression in PC [8,9]. To better clarify this topic, we performed a systematic literature review, analyzing the results of human tissue-based studies (immunohistochemistry, molecular analysis, etc.), experimental research (cell lines, mouse models), and clinical trials. We found that about 29% of acinar PCs, 7% of ductal PCs, and 46% of neuroendocrine carcinomas/tumors were PD-L1+ by immunohistochemistry, despite some variations in the positivity rate of different antibody clones, as well as potential limitations due to pre-analytical factors and inter-observer interpretation variability. We now present the results concerning the potential correlations of PD-L1 expression with the clinic–pathologic features of PC patients.

## 2. Materials and Methods

**Figure 1:**
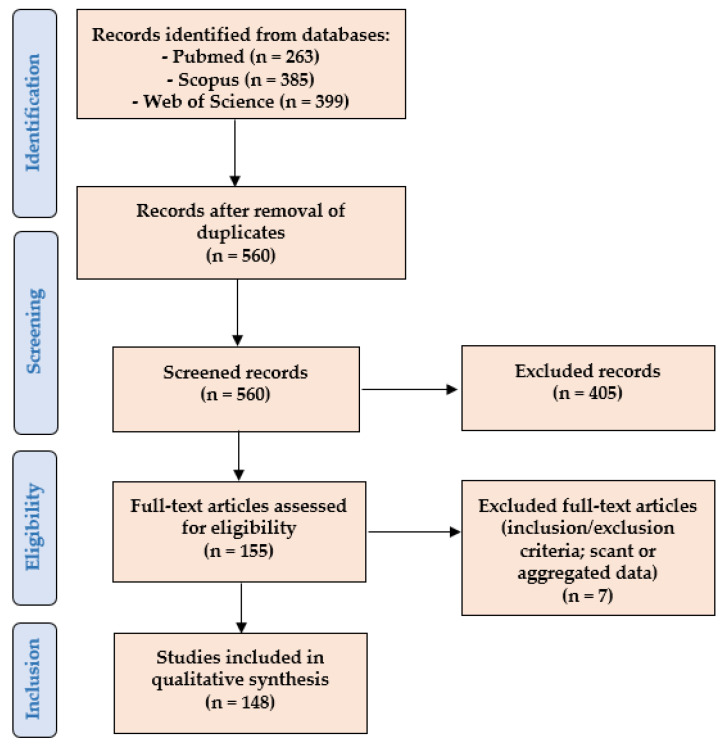
PRISMA flowchart of our systematic literature review.

We performed a systematic literature review according to the “Preferred Reporting Items for Systematic Reviews and Meta-Analyses” (PRISMA) guidelines (http://www.prisma-statement.org/ (accessed on 8 May 2021)) (Figure 1). 

Our goal was to summarize the data retrieved from the literature concerning the role of PD-L1 in PC, and to describe the clinic–pathologic features of the published cases. We answered the following “Population, Intervention, Comparison, Outcomes” (PICO) questions:

- Population: patients, tumor cell lines, or mouse models included in studies concerning the role of PD-L1 in PC;
- Intervention: any treatment;
- Comparison: no expected comparisons;
- Outcomes: patient status at last follow-up (no evidence of disease, alive with disease, dead of disease), response to therapy, biochemical recurrence-free survival, metastasis-free survival, cancer-specific survival, disease-free survival, clinical failure-free survival, overall survival, progression-free survival. For experiments on PC cell lines and mouse models: any reported effect on cancer and immune cell migration, proliferation, viability, growth, resistance/response to therapy, cytotoxic/anti-tumor activity, PD-L1 expression, and mice/cell lines survival.

Our retrospective observational study satisfied the following:

- Eligibility/inclusion criteria: experimental studies (tumor cell lines, mouse models) or clinic-pathologic studies on human patients concerning the role PD-L1 in PC;
- Exclusion criteria: non-prostatic tumors; non-carcinomatous histotypes; studies not examining PD-L1; uncertain diagnosis; review articles without new cases.

We searched for (PD-L1 AND (prostate OR prostatic) AND (adenocarcinoma OR adenocarcinomas OR cancer)) in the PubMed (all fields; 263 results; https://pubmed.ncbi.nlm.nih.gov (accessed on 8 May 2021)), Web of Science (Topic/Title; 399 results; https://login.webofknowledge.com (accessed on 8 May 2021)), and Scopus (Title/Abstract/Keywords; 385 results; https://www.scopus.com/home.uri (accessed on 8 May 2021)) databases. No limitations or additional filters were set. The bibliographic research ended on 8 May 2021. After the exclusion of duplicates, 560 records underwent first-step screening by 2 independent reviewers who checked the titles and abstracts of the articles to verify the satisfaction of the eligibility/inclusion criteria. They excluded clearly irrelevant studies, allowing articles of doubtful relevance to proceed to the following step in order to avoid the potential missing of pertinent papers. A total of 155 eligible articles were retrieved in full text format and read by 2 other authors (1) to look for additional relevant references, and (2) to confirm the satisfaction of the inclusion and exclusion criteria. After their evaluation, 7 papers were excluded, as they were unfit according to the inclusion criteria, or because they presented scant or aggregated data. Two other authors checked the extracted data, and 148 articles were finally included in our study [8,9,10,11,12,13,14,15,16,17,18,19,20,21,22,23,24,25,26,27,28,29,30,31,32,33,34,35,36,37,38,39,40,41,42,43,44,45,46,47,48,49,50,51,52,53,54,55,56,57,58,59,60,61,62,63,64,65,66,67,68,69,70,71,72,73,74,75,76,77,78,79,80,81,82,83,84,85,86,87,88,89,90,91,92,93,94,95,96,97,98,99,100,101,102,103,104,105,106,107,108,109,110,111,112,113,114,115,116,117,118,119,120,121,122,123,124,125,126,127,128,129,130,131,132,133,134,135,136,137,138,139,140,141,142,143,144,145,146,147,148,149,150,151,152,153,154,155].

Data collection was study-related (authors and year of study publication) and case-related (tumor stage at presentation, Grade Group, type of specimen, treatment, test methods and results of PD-L1 expression, follow-up and outcomes, experiment type). 

Statistical analysis: the collected data were reported as continuous variables (analyzed by ranges, mean and/or median values) or categorical variables (summarized by frequencies and percentages). Time-to-recurrence was the time from primary treatment to disease recurrence. The survival status was the time from primary treatment to the last follow-up. 

As there is a lot of data to discuss, we have divided the presentation of our results into different parts, focusing on different sub-topics. Here, we present the information concerning the investigated correlations of PD-L1 expression with clinic–pathologic features and outcome in PC patients.

## 3. Results

### 3.1. PD-L1 Expression and Grading Systems (Gleason Score, GS; Grade Group, GG)

About 29% of acinar PCs, 7% of ductal PCs, and 46% of neuroendocrine carcinomas/tumors were immunohistochemically positive for PD-L1 (further details are available in the other parts of our review). Data of non-acinar histotypes are too scant to discuss relevant correlations, so the following considerations will be related only to acinar PCs. 

The association of PD-L1 expression with GG/GS is controversial, partly because the published series typically included PCs of variable GG/GS without specifying the positivity rates of each GG/GS subgroup. 

In the series of Xian et al. (*n* = 279) [43], PD-L1 expression was significantly associated with high GG (*p* = 0.0001); the correlation was still valid when patients were divided into 5 GGs (*p* = 0.0010). GS6 (GG1) PCs revealed scattered PD-L1+ cells, GS7 (GG2-3) PCs were weakly positive, and GS9 (GG5) PCs showed strong PD-L1 positivity. Haffner et al. (*n* = 508; *p* = 0.08) [66] and Calagua et al. (*n* = 351; *p* = 0.013 for 1% cut-off of PD-L1 positivity, GG4-5 vs. GG1-3) [75] also found increased PD-L1 expression in high-grade tumors. In the study of Shim et al. (*n* = 171) [12], GG positively correlated with membranous PD-L1 expression, being inversely associated with nuclear PD-L1 positivity; there was intratumoral heterogeneity in PD-L1 expression among different GSs, and no significant correlation between membranous and nuclear PD-L1 expression was found.

On the other hand, Iacovelli et al. (*n* = 32) [39] reported that there was a higher incidence of GS ≥ 8 (GG4-5) PCs among PD-L1-negative than PD-L1-positive tumors (100% vs. 76.9%; *p* = 0.037). In the series of Najjar et al. [84] (*n* = 129), 6/7 (86%) PD-L1+ cases showed “low” (not otherwise specified) GG, without reaching statistical significance.

Scimeca et al. (*n* = 50) [48] and Obradovic et al. (*n* = 29) [29] did not find any correlation between GG and PD-L1 expression in cancer cells. Moreover, Lindh et al. [41] confirmed this result for either tumor cells or tumor-infiltrating lymphocytes (TILs), both in ductal (*p* = 0.42) and acinar PC histotypes (*p* = 0.29). Finally, Baas et al. [80] (*n* = 25; subjective semiquantitative score) did not identify any significant association between PD-1, PD-L1, and CD3 with GG.

### 3.2. PD-L1 Expression and Tumor Stage: pT

In the radical prostatectomy series of Xian et al. (*n* = 279) [43], PD-L1 expression in tumor cells was significantly associated with age ≥65 years, body mass index ≥30, and advanced tumor stage (T1/2 vs. T3/4, *p* = 0.0037). Calagua et al. (*n* = 351) [75] found that patients with PD-L1+ PCs had higher serum PSA levels, high tumor stage, and rate of margin positivity on radical prostatectomy specimens. Accordingly, in the series of Sharma et al. (*n* = 220) [27,35], PD-L1 was more frequently expressed in PCs at a high pT stage (pT2, 10.8% vs. pT3/4, 20.4%; *p* = 0.072; pT2/3a, 11.4% vs. pT3b/4, 31.6%; *p* = 0.013). 

In the study of Shim et al. (*n* = 171) [12], tumor stage was significantly higher in the group of PCs showing nuclear staining of PD-L1. Conversely, only pre-treatment PSA (but neither GG nor stage) was significantly higher in tumors revealing conventional membranous PD-L1 expression. However, these data were not significant on multivariate analyses. Other smaller series (Obradovic et al.: 29 cases [29]; Wagle et al.: 21 cases [28]) did not find any correlations between PD-L1 expression and stage.

### 3.3. PD-L1 Expression and Tumor Stage: Lymph Node Status (pN) and Distant Metastases (pM)

Shaw et al. (*n* = 91) [36] reported that PD-L1 positivity was more common in “high-risk” localized PCs (13/50 cases, 26%) and metastatic PCs (7/41 cases, 17%). 

In some studies, PD-L1 expression was more often seen in PCs with lymph node metastases, sometimes reaching a statistically significant association: Xian et al. (*n* = 279; *p* = 0.0294) [43]; Sharma et al. (*n* = 220; pN0 10.1% vs. pN1 27.3%; *p* = 0.086) [27,35]. Iacovelli et al. [39] found that lymph node metastases were more frequent in PCs expressing PD-L1 in ≥1% of tumor cells (93% vs. 65%; *p* = 0.05; *n* = 32); this difference became significant when considering a ≥5% cut-off for PD-L1 positivity (60% vs. 40%; *p* = 0.044). No difference in PD-L1 expression was found in patients with high vs. low metastatic disease volume (based on CHAARTED classification: visceral metastases or ≥4 bone lesions with ≥1 beyond the vertebral bodies and pelvis), or among the different metastatic sites. 

In the series of Petitprez et al. (*n* = 51) [77], patients with PD-L1+ PCs had a four-fold increased risk of experiencing distant metastases: the number of positive lymph nodes (*p* = 0.004) significantly differed between patients with PD-L1+ and PD-L1− tumors. However, Baas et al. [80] did not find any significant association concerning PD-1, PD-L1, and CD3 expression in metastases (*n* = 25). 

The PD-L1 positivity rate of metastatic PCs was usually analyzed on the primary tumor (typically by testing the radical prostatectomy specimen or the biopsies). In other cases, it was unclear which sample (primary tumor vs. metastases) was tested. The few clear studies on the variability of the PD-L1 positivity rate between primary and metastatic-matched PC samples yielded interesting results. In their series, Fankhauser et al. [74] found that all the five PD-L1+ primary PCs showed no PD-L1 expression in bone (*n* = 10), brain (*n* = 1), lung (*n* = 1), or lymph node (*n* = 1) metastases. There was also variability in the PD-L1 positivity rate among metastases of castration-resistant PCs (CRPCs) to different sites in the autopsy cohort of Haffner et al. [66]: CRPC metastases showed higher rates of PD-L1 expression, namely >31% and up to 11% of cases with PD-L1 expression in ≥1% and ≥5% of PC cells, respectively, regardless of the tumor site [66]. Finally, Ihle et al. reported a higher PD-L1 positivity rate in blastic (*n* = 5) than in lytic bone metastases (*n* = 10) [31].

### 3.4. PD-L1 Expression and Overall Survival

The potential correlation of PD-L1 with overall survival (OS) was rarely investigated. The large series of Nagaputra et al. (*n* = 211) [67] disclosed a trend toward poorer OS in AR-/PD-L1- PCs (*p* = 0.055). 

Lin et al. [154] recently reported a clinical trial involving 206 men with previously untreated metastatic CRPCs (mCRPCs) harboring high microsatellite instability and PD-L1 staining (combined positive score, CPS ≥ 1): 106/206 patients received pembrolizumab alone (PA-group), while pembrolizumab + enzalutamide (ENZ) were administered to 100/206 men (PE-group). PD-L1 CPS was 1–20 in 64%, 20–50 in 22%, and 50–100 in 14% of PCs of the PE group, accounting for 1–20 in 63%, 20–50 in 22%, and 50–100 in 15% of PA patients (*p* = 0.872). Among the PE and PA groups, the median OS was 28.6 vs. 21.3 months for a PD-L1 CPS ≥50 (*p* = 0.001), 26.6 vs. 19.4 months for a CPS ≥20 (*p* = 0.001), and 21.4 vs. 16.8 months for a CPS ≥1 (*p* = 0.001); the benefit from PE therapy seemed more evident for higher PD-L1 expression in tumor cells (indicating an anti-tumor immune response).

Conversely, Iacovelli et al. [39] found no difference in median OS between PD-L1− and PD-L1+ patients (43.8 vs. 29.6 months; *n* = 32; *p* = 0.88), also in men who received androgen deprivation therapy as first-line treatment (55.16 vs. 29.6 months; *p* = 0.89). In keeping with this, Xian et al. [43] found no statistically significant correlation between OS and PD-1 or PD-L1 expression (*n* = 279). In the large series of Zhao et al. (*n* = 9393), the authors reported on the lack of association between PD-L1 and OS, yet the method of PD-L1 testing and scoring was not completely clear [47].

### 3.5. PD-L1 Expression and Tumor Recurrence

The potential correlation of PD-L1 expression with clinical outcome—in terms of biochemical recurrence-free survival (BCRFS), metastasis-free survival (MFS), cancer-specific survival (CSS), disease-free survival (DFS), and clinical failure-free survival (CFFS)—was rarely investigated, reporting conflicting results:

**Positive correlation:**

- According to some authors [89,93], PD-L1 was an independent prognostic factor of biochemical recurrence (BCR) (*p* = 0.007; *n* = 820) on multivariate analysis (also including tumor stage, surgical margins, GS/GG, and preoperative PSA). PCs were scored as having low vs. high PD-L1 expression, using a median value as cut-off. PD-L1^high^ expression with or without concomitant aberrant *CXCL12* methylation (mCXCL12) was significantly associated with shorter BCRFS (*p* = 0.005). PD-L1^low^ cases showed the longest BCRFS (mean 112 months), while mCXCL12^medium^ expressors showed best BCRFS rates among PD-L1^high^ cases (mean 107 months).
- Li et al. (*n* = 127) [44] reported shorter median BCRFS for PD-L1^high^ PCs (18.5 vs. 72.5 months): PD-L1^high^ was an independent predictor for time-to-BCR on a multivariate analysis (*p* = 0.016), being associated with lower BCRFS both in PD-1+ (*p* = 0.0193) and PD-1– cases (*p* < 0.0001). In localized PCs, the median BCRFS was dramatically lower in PD-L1^high^ PCs (16 vs. 72.5 months; *p* < 0.0001), not being associated with the PD-1 status. In metastatic patients, BCRFS was not significantly correlated with the PD-L1/PD-1 status. PD-L1^high^ and PD-1 negativity were significantly associated with lower PSA density (*p* = 0.010 and *p* = 0.033, respectively).
- Zhou et al. found notably higher PD-1/PD-L1 expression in patients with recurrent PCs (*p* = 0.016; *n* = 122) [120].
- In a smaller series (*n* = 45), the median MFS was shorter in PD-L1+ patients (49 vs. 68 months; *p* = 0.090): multivariate analysis confirmed the independent predictive value of expression and hyperexpression of PD-L1 in tumor cells for MFS (*p* = 0.025, *p* = 0.032) and CSS (*p* = 0.097, *p* = 0.065) [38].
- Petitprez et al. (*n* = 51) [77] found that PCs with ≥1% of PD-L1+ tumor cells had shorter MFS (*p* = 0.008). PD-L1+ and/or CD8^high^ PCs showed a higher risk of recurrence after radical prostatectomy.

**Negative, unclear, or absent correlation**:

- In the large series of Zhao et al. (*n* = 9393) [47], PD-L1 expression was not associated with prognostic outcomes (BCRFS, MFS, CSS, OS). Conversely, PD-L2 was associated with worse BCRFS, MFS, CSS, immune-related pathways on gene set enrichment analyses and radiation response pathways: it predicted the response to postoperative radiotherapy on multivariate analysis (*p* = 0.03). However, data were obtained from seven previously published radical prostatectomy cohorts and the method of PD-L1 testing and scoring was not completely clear.
- Some studies [42,79] reported that PD-L1 positivity in tumor cells did not reach statistical significance for predicting BCR or clinical failure, but there was a trend toward a negative association between PD-L1 expression and BCR (*n* = 402; *p* = 0.078). On multivariate analysis, PD-1^high^ expression in intratumoral lymphocytes was a negative independent prognostic factor for CFFS (*p* = 0.025).
- Nagaputra et al. (*n* = 211) [67] reported that PCs negative for both AR and PD-L1 disclosed unfavorable DFS (*p* = 0.037), but there was no significant impact on BCR.
- By using fluorescent immunohistochemistry on tissue microarrays (TMAs), Vicier et al. [25] analyzed the total density of PD-L1 positivity in 109 PCs: men with CD8^low^ and/or PD-L1^high^ expression had significantly shorter time-to-BCR (median 3.5 years vs. not reached) and MFS (median 10.8 vs. 18.4 years); however, CD8^low^ or PD-L1^high^ alone were not independent predictors of BCR or MFS on multivariate analysis.
- Obradovic et al. found that PD-L1 expression negatively correlated with BCR or metastatic recurrence, but it was not predictive of time-to-recurrence (*n* = 29) [29].
- Baas et al. (*n* = 25) [80] did not find any significant association between PD-1, PD-L1, or CD3 with BCR.
- Both membranous and nuclear PD-L1 expression were not predictive of BCRFS on univariate and multivariate analyses in the study of Shim et al. (*n* = 171) [12].

Mo et al. [50] found that a higher density of PD-L1+ tumor-associated nerves was significantly associated with BCR (*p* = 0.016), representing an independent prognostic factor of BCR on univariate and multivariate analyses (*p* = 0.018, *p* = 0.042) (*n* = 80). However, PD-L1 positivity in stromal cells did not reach statistical significance for predicting BCR or clinical failure in another series (*n* = 402) [42,79].

### 3.6. PD-L1 Expression and Treatment

The positivity rate of PD-L1 seemed higher in CRPCs (440/904, 49%) [8,9,13,17,21,27,35,38,50,55,59,62,66,74,90,92,98,99,100] as compared to hormone-sensitive PCs (44/254, 17%): PD-L1 was usually tested on primary tumors that probably had not yet undergone hormonal therapy as this information was not always available [27,32,35,39]. 

Matveev et al. [37] described 10/35 (28.6%) PD-L1+ metastatic hormone-naïve cases: median time to castration resistance was significantly lower in PD-L1+ patients (21.44 vs. 49.12, *p* = 0.006). Multivariate analysis confirmed the independent prognostic value of PD-L1 positivity, resulting in decreased time to castration resistance (*p* = 0.002) also in patients with low-volume metastatic disease (*p* = 0.005). Li et al. (*n* = 127) [44] related the high PD-L1 expression to the worse prognosis of adjuvant hormonal therapy. In another study [94], none of the 11 hormonally treated samples were PD-L1+. Mo et al. [50] described that 1/73 (1%) regionally localized PCs (RLPCs) and 0/7 (0%) CRPCs expressed PD-L1 on tumor cells; 69/73 (94.5%) RLPCs and 2/7 CRPCs were positive for PD-L1 in the tumor-associated stroma (nerve branches), supported by co-localization with axonal marker PGP9.5.

Gao et al. reported significantly greater protein expression of PD-1, PD-L1, and VISTA in PCs after ipilimumab therapy (*n* = 17) [81]. In the series of Obradovic et al. (*n* = 29) [29], both the cyclophosphamide/GVAX/degarelix and the degarelix-alone groups (*n* = 29; 1:1 randomization) led to a significant increase in intratumoral CD8+ T-cells and PD-L1 expression: GVAX vaccine did not significantly increase CD8+ T cell density, but the authors suggested that GVAX-induced infiltrating immune cells may promote PD-L1 upregulation. Conversely, Calagua et al. [75] found that neoadjuvant androgen deprivation therapy (neo-AAPL; *n* = 44) was associated with reduced CD8+ TILs; these PCs showed a trend toward decreased PD-L1 expression as compared to untreated tumors (7% vs. 21%). 

Vardaki et al. reported that plasma exosomes of patients with an unfavorable outcome had higher levels of PD-L1 as compared to men with a favorable prognosis (Western blot analysis, Luminex multiplex array); these changes were Radium-223-dependent without differences in the immune checkpoint modulators upon cabazitaxel treatment [11]. 

Information concerning the details of clinical trials and patients’ responses to immunotherapies is discussed in another part of our systematic literature review (see Section 2).

### 3.7. Evaluation of PD-L1 Expression in Tumor Tissue: Real-Time Polymerase Chain Reaction (PCR)

PD-L1 expression was usually investigated by immunohistochemistry, while few studies correlated the abovementioned clinic–pathologic features and outcome variables with the data of the PD-L1 status derived from PCR analysis. Some authors found that normal tissue showed lower levels of PD-L1 RNA [22] and PD-L1 promoter methylation (mPD-L1) [86] as compared to PC samples. Xiong et al. reported that MLL3 and PD-L1 RNA levels were higher in PC metastases than in primary PC tumors, positively correlating with PSA levels (not with GG, age, or stage) [56]. In a large series [86], high mPD-L1 (*p* = 0.008) and high PD-L1 protein expression (pePD-L1) (*p* = 0.002) (analyzed as continuous variables) both correlated to shorter BCRFS on multivariate analysis (compared to pePD-L1^low^/mPD-L1^low^); these results were not confirmed in the validation cohort. Patients with pePD-L1^high^/mPD-L1^low^ or pePD-L1^low^/mPD-L1^high^ showed intermediate BCRFS. PD-L1 DNA methylation was associated with pT stage (*p* < 0.001) and GG (*p* = 0.001). 

## 4. Discussion

The interaction between PD-1 and its ligand PD-L1 plays a pivotal role in balancing the peripheral tolerance with self-tolerance, and can be exploited by cancer cells in order to escape the immune surveillance, resulting in tumor proliferation and progression. As a consequence, the discovery of the PD-1/PD-L1 pathway and its functions has reshaped our way of regarding tumor immunology and treatment [156]. Since then, several clinical trials have been performed to assess the outcome of cancer patients treated with agents targeting the PD-1/PD-L1 pathway [156]. Recently, an anti-PD-1 antibody (pembrolizumab) revealed promising therapeutic activity in a phase II trial, and the 2021 NCCN guidelines have allowed its administration in selected PC cases [4,9].

Data have suggested that PD-L1 immunohistochemical expression is a potential predictive biomarker for response to immune checkpoint inhibitors, at least in some tumor types [100]. However, the role of PD-L1 detection in selecting patients amenable to immunotherapy is still debated [157]. 

Increased expression of PD-L1 is a common finding in tumor cells from various sites, including lung, ovary, kidney, pancreas, etc. [158,159,160,161,162,163,164]. The potential prognostic value of PD-L1 immunohistochemical expression has been investigated in different malignancies, often correlating with unfavorable clinic–pathologic features [164,165,166,167,168,169,170]. 

In our review, the PD-L1 positivity rate was usually higher in PCs than in benign tissues, globally accounting for 29% of acinar PCs, 7% of ductal PCs, and 46% of neuroendocrine carcinomas/tumors, despite some limitations in the immunohistochemical analysis and interpretation, as well as in the variability concerning the positivity rates among the different series [8,9,10,11,12,13,14,15,16,17,18,19,20,21,22,23,24,25,26,27,28,29,30,31,32,33,34,35,36,37,38,39,40,41,42,43,44,45,46,47,48,49,50,51,52,53,54,55,56,57,58,59,60,61,62,63,64,65,66,67,68,69,70,71,72,73,74,75,76,77,78,79,80,81,82,83,84,85,86,87,88,89,90,91,92,93,94,95,96,97,98,99,100,101,102,103,104,105,106,107,108,109,110,111,112,113,114,115,116,117,118,119,120,121,122,123,124,125,126,127,128,129,130,131,132,133,134,135,136,137,138,139,140,141,142,143,144,145,146,147,148,149,150,151,152,153,154,155].

As other previously published meta-analyses on this topic, our systematic literature review has inherent limitations [8,9,10,11,12,13,14,15,16,17,18,19,20,21,22,23,24,25,26,27,28,29,30,31,32,33,34,35,36,37,38,39,40,41,42,43,44,45,46,47,48,49,50,51,52,53,54,55,56,57,58,59,60,61,62,63,64,65,66,67,68,69,70,71,72,73,74,75,76,77,78,79,80,81,82,83,84,85,86,87,88,89,90,91,92,93,94,95,96,97,98,99,100,101,102,103,104,105,106,107,108,109,110,111,112,113,114,115,116,117,118,119,120,121,122,123,124,125,126,127,128,129,130,131,132,133,134,135,136,137,138,139,140,141,142,143,144,145,146,147,148,149,150,151,152,153,154,155]. First, there is a wide heterogeneity among studies in terms of antibody clones, testing features, scoring methods, sample types, inclusion/exclusion criteria, tumor clinic–pathologic features and treatment, which prevents a standardization of results. Tissue microarrays allow for the examination of only a small area of tumor tissue and might further hamper the reliability of the scoring [120]. Moreover, inter-observer variability in the interpretation of the immunohistochemical results may occur, and a further confounding factor is the detection of PD-L1 expression on both tumor cells and lymphocytes (especially in cases associated with significant inflammation). PD-L1 positivity in other inflammatory (macrophages, plasma cells) or stromal cells may represent another pitfall in assessing a definite rate. Finally, the vast majority of published series was retrospective and the number of studies investigating PD-L1 expression in PC is still limited; therefore, further large-cohort studies are warranted.

Indeed, only few studies have assessed the potential prognostic correlation of PD-L1 expression with clinic–pathologic features in PC cases.

The Gleason score provides vital information for guiding the management and prognostication of PC patients [171]. In 2013, Epstein et al. used the criteria of the Gleason score to propose five prognostic risk categories (Grade Groups), applying a scale of 1–5: PCs with the most favorable features (Gleason score 6) were classified as Grade Group 1. The distinct risk of BCR based on each Grade Group has been confirmed in large multicenter studies, as for the risk of death due to PC [172,173].

The association of PD-L1 expression with GG was sometimes investigated [12,29,39,41,43,48,66,75,80,84]. Despite some studies reporting variable or controversial results, the relatively larger series revealed increased PD-L1 expression in high-grade tumors [12,43,66,75]. This correlation was occasionally statistically significant, still being valid when patients were divided into five GGs (Xian et al., *n* = 279, *p* = 0.0010) [43].

The different scoring systems/cut-offs for PD-L1 expression, the variability of tested sample types, the various antibody clones, and other biases may interfere with this potential correlation [8,9,10,11,12,13,14,15,16,17,18,19,20,21,22,23,24,25,26,27,28,29,30,31,32,33,34,35,36,37,38,39,40,41,42,43,44,45,46,47,48,49,50,51,52,53,54,55,56,57,58,59,60,61,62,63,64,65,66,67,68,69,70,71,72,73,74,75,76,77,78,79,80,81,82,83,84,85,86,87,88,89,90,91,92,93,94,95,96,97,98,99,100,101,102,103,104,105,106,107,108,109,110,111,112,113,114,115,116,117,118,119,120,121,122,123,124,125,126,127,128,129,130,131,132,133,134,135,136,137,138,139,140,141,142,143,144,145,146,147,148,149,150,151,152,153,154,155]. In particular, the different types of analyzed specimens may interfere not only with the PD-L1 positivity rate (especially in studies using TMAs) but also with the assessment of GG [29]. In fact, PD-L1 expression may be focal/heterogeneous, while GG could increase (or decrease) from the biopsy to the radical prostatectomy (RP) or metastatic (MTS) specimens of the same patients. Indeed, tumor nodules of variable GG may be present in the same prostate, and/or the relative percentages of the different Gleason patterns identified in a biopsy may change in RP/MTS samples. Higher Gleason patterns may be identified only in RPs/MTS, being absent in previous biopsies [174]. The abovementioned studies of Xian et al. and Calagua et al. analyzed RP specimens [43,75], while other series included biopsies [12,80] or TMAs variably assessing ≥1 specimen types (biopsies, RPs, autopsy material, transurethral resections of prostate) [29,39,41,48,66]. The promising results of Xian et al. should be validated in larger series that include different specimen types. Additionally, attention may be given to different staining (nuclear vs. membranous) patterns, as reported by some authors [12].

The correlation between PD-L1 and Ki-67 expression, the latter being a well-known marker of cell proliferation, provides further evidence of the adverse prognostic role of PD-L1 [93].

Some series favored the fact that PD-L1 positivity may also be correlated to a more aggressive tumor stage and/or metastatic behavior. Except for one study (*n* = 171) [12], large cohorts showed that PD-L1 expression was higher in the advanced pT tumor stages (Xian et al., *n* = 279; Calagua et al., *n* = 351) [43,75]; conversely, smaller series (Obradovic et al.: 29 cases [29]; Wagle et al.: 21 cases [28]) did not find any correlation. Calagua et al. [75] also reported that patients with PD-L1+ PCs had higher serum PSA levels and rate of margin positivity on RP specimens. Moreover, PD-L1 expression was more often seen in PCs with lymph node metastases in some studies, occasionally reaching a statistically significant association [27,35,43]. Larger multicenter series have to verify these data, as well as the efficacy of different cut-off percentages of PD-L1 immunohistochemical positivity to identify cases with metastatic potential. In fact, a study including only 32 cases [39] found that the association of lymph node metastases became significant when considering a ≥5% cut-off for PD-L1 expression (60% vs. 40%; *p* = 0.044), while a cut-off ≥1% did not reach statistical significance. 

Pre-analytical variables, tumor clone heterogeneity, and the frequently patchy/focal PD-L1 positivity in PC tumor cells may also question the utility and reliability of PD-L1 immunohistochemical testing in PCs in assessing eligibility for immunotherapy administration [8,9,10,11,12,13,14,15,16,17,18,19,20,21,22,23,24,25,26,27,28,29,30,31,32,33,34,35,36,37,38,39,40,41,42,43,44,45,46,47,48,49,50,51,52,53,54,55,56,57,58,59,60,61,62,63,64,65,66,67,68,69,70,71,72,73,74,75,76,77,78,79,80,81,82,83,84,85,86,87,88,89,90,91,92,93,94,95,96,97,98,99,100,101,102,103,104,105,106,107,108,109,110,111,112,113,114,115,116,117,118,119,120,121,122,123,124,125,126,127,128,129,130,131,132,133,134,135,136,137,138,139,140,141,142,143,144,145,146,147,148,149,150,151,152,153,154,155]. As for other tumor-types, generous cut-offs and scoring criteria (≥1%, CPS) may sometimes easily allow the pathologist to consider a case as positive; thus, it can be debated if the efficacy of the administered drug is related to a very focal PD-L1 immunohistochemical expression. Similarly, the potential correlation of a very limited PD-L1 positivity with outcome variables (GG/GS; stage; OS; progression-free survival; PFS, etc.) could be questionable. Moreover, the few studies testing matched primary PC samples and PC metastases of the same patients revealed different PD-L1 positivity rates, which can also vary among the different lymph node or distant metastatic sites. In an autopsy cohort, CRPC metastases showed higher rates of PD-L1 expression, regardless of tumor site, and there was some variability in PD-L1 staining among the CRPC metastases to different sites [66]. Autopsy specimens may undergo various degenerative changes that could influence the immunohistochemical, result but these data were also confirmed in some surgical specimens series, showing a better preserved status of the analyzed material. For example, Fankhauser et al. [74] found that all five PD-L1+ primary PCs of their series showed no PD-L1 expression in distant metastases. Further studies are required, also to verify which sample type could be the best predictor of clinical and treatment outcome.

The bone is one of the more frequently involved sites of PC metastases [1]. Despite there is still a debate on this topic, overfixation, type of decalcifier, and duration of decalcification may influence the immunohistochemical analysis of PD-L1, causing false-negative results or reduced expression by the tested cells [175,176,177]. Moreover, the various tumor types and PD-L1 antibody clones may be variably influenced by these factors [177]. This topic seemed not clearly studied in large series of PC cases. Ihle et al. interestingly reported that PD-L1 expression was higher in blastic (*n* = 5) than in lytic bone metastases (*n* = 10) [31]. 

Another support for the potential aggressive behavior of PD-L1+ PCs is that, globally, the positivity rate of PD-L1 seemed higher in CRPCs (440/904, 49%) [8,9,13,17,21,27,35,38,50,55,59,62,66,74,90,92,98,99,100] than in hormone-sensitive PCs (44/254, 17%). PD-L1 was usually tested on primary tumors that probably had not yet undergone hormonal therapy [27,32,35,39]. Some series found that PD-L1 positivity was associated with decreased time to castration resistance [37,44]. However, these limited data require further confirmation that these patients might benefit from immunotherapy.

Treatment with ipilimumab (an anti-CTLA-4 antibody) may increase the protein expression of PD-1, PD-L1, and VISTA in PCs [81], while cyclophosphamide/GVAX/degarelix or degarelix alone may increase intratumoral CD8+ T-cells and PD-L1 expression [29]. GVAX-induced infiltrating immune cells may promote PD-L1 upregulation. Conversely, neoadjuvant androgen deprivation therapy may reduce the number of CD8+ TILs, with a trend toward decreased PD-L1 expression by PC tumor cells (compared with untreated tumors: 7% vs. 21%) [75].

Despite the potential association of PD-L1 expression with aggressive clinic–pathologic features (higher GG and stage, castration resistance), a correlation of PD-L1 with OS was rarely investigated, producing conflicting results. The large series of Nagaputra et al. (*n* = 211) [67] disclosed a trend toward poorer OS in AR-/PD-L1- PCs (*p* = 0.055). In another series, the benefit in OS from treatment with pembrolizumab and enzalutamide seemed more evident for higher PD-L1 expression in tumor cells [154]. Conversely, other studies, including some large series, found no difference in OS between PD-L1+ and PD-L1- cases despite some limitations [39,43,47]. Further data are needed.

Similarly, few studies (sometimes including large series) analyzed the potential correlations of PD-L1 expression with clinical outcomes related to tumor recurrence. Some evidence suggested the potential association of PD-L1 positivity with reduced BCRFS/MFS/CSS [38,56,86,89,93], thus arguing that the disruption of anti-tumor immunity exerted by PD-L1 expressed on cancer cells is an underlying mechanism of tumor recurrence [178]. Several mechanisms of evasion of PD-L1+ tumor cells from T-lymphocytes immune reaction have been proposed, including the induction of apoptosis and anergy, and increasing the production of immunosuppressive cytokines [179]. However, other series did not reveal a clear correlation of PD-L1 expression with outcomes related to tumor recurrence: additional investigations are required to verify the controversial prognostic role of PD-L1, as limitations were present in previous studies. Other factors and molecular biomarkers may interfere, allowing for the further stratification of patients (*CXCL12* methylation, *MLL3* status, etc.) [56,89,93].

Few papers investigated the potential association of PD-L1 expression with clinical outcome on blood samples. Vardaki et al. (*n* = 25) found that the plasma exosomes of patients with an unfavorable prognosis had higher levels of PD-L1 compared to men with a favorable outcome: these changes were Radium-223-dependent, without differences in the immune checkpoint modulators upon Cabazitaxel treatment [11]. Satelli et al. (*n* = 30) [91] reported that nuclear PD-L1 expression in circulating tumor cells was significantly associated with worse PFS, while no correlation with OS was found. Conversely, increased PD-L1 expression was associated with longer time-to-disease progression by Rekoske et al. (*n* = 17) [147]. Finally, in the study of Wang et al. (*n* = 190) [23], serum PD-L1 levels did not correlate with BCRFS or PFS, while serum PD-L2 levels were significantly associated with BCRFS (*p* < 0.05) and PFS.

As they are becoming increasingly important in health care, systematic literature reviews conducted according to the PRISMA guidelines include an evidence-based minimum set of items for reporting (http://www.prisma-statement.org/ (accessed on 8 May 2021)). We have performed our research according to these guidelines, as previously described in various contexts in which they could play a key role in the dissemination of knowledge [180,181,182,183,184,185,186,187,188,189,190,191,192,193,194,195,196,197,198,199,200,201,202,203,204,205,206,207,208,209,210,211,212]. To better present our results and to clarify the role of PD-L1 in PC, we have divided our results into different articles, highlighting relevant subtopics. In the other papers, the readers will find further information about the following subjects: PD-L1 immunohistochemical expression in PC with a discussion of pre-analytical and interpretation variables; correlations of PD-L1 expression with the status of mismatch repair system, *BRCA*, *PTEN,* and other main genes in PC; PD-L1 intracellular signaling pathways in PC and the regulation of the tumor microenvironment; pre-clinical models (cell lines, mouse models) and experimental treatments affecting PD-L1 expression in PC cells; genetic and epigenetic regulation of PD-L1; PD-L1 expression in liquid biopsies; results of clinical trials, etc. [213,214,215,216].

## 5. Conclusions

A number of studies have investigated the potentially prognostic and predictive role of PD-L1 in PC. PD-L1 expression by tumor cells may be related to the clinic–pathologic features of adverse outcome, including advanced tumor stage (high pT, lymph node, or distant metastases), positivity of surgical margins, high GS/GG, and castration resistance status. Different PD-L1 positivity rates may be observed in primary PCs and various metastatic sites of the same patients. Over fixation, type and duration of decalcification, and PD-L1 antibody clones may influence the immunohistochemical analysis of PD-L1 on bone metastases from PC.

The positivity rate of PD-L1 seemed higher in CRPCs (49%) as compared to hormone-sensitive PCs (17%), and some series found that PD-L1 positivity was associated with decreased time to castration resistance. Treatment with ipilimumab, cyclophosphamide/GVAX/degarelix, or degarelix alone may increase PD-L1 expression; conversely, other studies found that neoadjuvant androgen deprivation therapy may reduce PD-L1 expression by PC tumor cells.

The correlation of PD-L1 expression with OS and outcomes related to tumor recurrence was rarely investigated: the few studies produced conflicting results and sometimes showed limitations. Further well-designed, multicenter studies on larger, selected cohorts are needed to elucidate the points highlighted by our systematic literature review, also including the potentially different prognostic and predictive roles of PD-L1 expression in various groups of PC patients (castration-sensitive vs. castration-resistant, limited-stage vs. metastatic, etc.). The testing and scoring of PD-L1 should be standardized. 

Most of the available data were related to the acinar type of PC. Additional studies are required, especially for rare PC histotypes such as ductal and neuroendocrine carcinomas, with the latter showing a higher PD-L1 positivity rate.

Pembrolizumab administration was allowed by the 2021 NCCN guidelines as a second-line (or beyond) therapy in selected mCRPC patients with tumors showing high microsatellite instability or mismatch repair system deficiency, progressing after docetaxel and/or novel hormone therapy [4]. However, these guidelines do not currently report the need for an immunohistochemical evaluation of PD-L1 in PCs. Indeed, some PD-L1+ tumors may not respond to immunotherapy, while other PD-L1- cases can. Moreover, PD-L1 immunohistochemical positivity is frequently focal (usually assessed for ≥1% of stained cells), and it could not be a reliable predictor of the tumor responsiveness to immunotherapy. Finally, it is unclear which sample is best for testing as the positivity rate varies among different types of specimens. Future studies should clearly provide details of the tested cases. PD-L1 immunohistochemical expression may be confirmed as not useful (at least alone) to select PC patients for the inclusion in clinical trials. Other immune checkpoints or targets that may be clinically relevant in PCs should be investigated, supporting the trial selection and/or allowing for the development of more effective combinatorial strategies for treatment; multiple targeting may improve the immunogenicity of PC cells and of the tumor microenvironment.
